# What are effects of a spaced activation of virtual patients in a pediatric course?

**DOI:** 10.1186/1472-6920-13-45

**Published:** 2013-03-28

**Authors:** Esther M Maier, Inga Hege, Ania C Muntau, Johanna Huber, Martin R Fischer

**Affiliations:** 1 University Children’s Hospital Salzburg, Salzburg, Austria; 2 Lehrstuhl für Didaktik und Ausbildungsforschung in der Medizin, Klinikum der Ludwig-Maximilians-University, Munich, Germany; 3 Dr. von Hauner Children’s Hospital, Ludwig-Maximilians-University, Munich, Germany

**Keywords:** Virtual patients, Pediatrics, Spaced activation, E-learning

## Abstract

**Background:**

Virtual patients (VPs) have a long tradition in the curriculum of the medical faculty at the Ludwig-Maximilians-University (LMU) Munich. However, the pediatric VPs were not well integrated into the curriculum and hardly used by students.

**Methods:**

Therefore we created and implemented a self-contained E-learning module based on virtual patients (VPs), which was embedded into the pediatric curriculum.

Students taking this course were divided into two groups. For Group A the virtual patients were activated in a timed order (“spaced activation”), whereas Group B could work on all VPs from the beginning.

We investigated the performance of these two groups concerning usage pattern including number of sessions and session duration, score on questions integrated into the VP and results of the intermediate exam.

**Results:**

The integration of the VPs into the pediatric course was successful for both groups. The usage pattern for the spaced activation turned out to be more balanced, however we did not find any significant differences in the results of the intermediate exam, the score on questions included in the VPs nor in the time students spent working on the VPs.

**Conclusions:**

Our study showed that the spaced activation led to a more balanced VP usage pattern with a lower peak of sessions at the end of the course. Further studies will have to investigate whether a spaced activation of VPs leads to favorable long-term learning outcomes.

## Background

Over the past 15 years the use of E-learning modules has become more and more popular at the medical faculty at the LMU Munich. Especially in internal medicine, the use of Virtual Patients (VPs) has a long tradition [1].

In the past for the pediatric course six VPs were available for the students. These VPs were neither connected to the learning objectives taught in the pediatric course nor did they form a self-contained learning unit like for example the CLIPP project in the USA [2]. Covering mainly rare conditions such as malaria, the pediatric VPs were hardly used.

Therefore, these VPs were replaced by a self-contained E-learning module which was integrated into the pediatric course and covered relevant learning objectives of the curriculum.

Former studies have shown that several aspects are important for successful integration of E-learning into a curriculum. The effective implementation of VPs, however, needs to be clarified according to a meta-analysis by Cook et al. [3].

A combination of obligatory and optional exam relevant VPs, that have been designed to cover the relevant curricular objectives has been proven successful in a previous study at our faculty [4]. Several studies have revealed that students tend to work on exam relevant E-learning modules a few days prior to their exam for the first time, instead of repeating the modules before the exam [4]. A study performed by Dünne et al. indicates that a short interval between learning and an exam correlates with a better performance in the exam [5]. On the other hand, this lack of repetition might lead to lower long-term knowledge retention [6].

In addition, a review by Roher et al. concludes that the so-called spacing effect improves long-term knowledge retention [7]. Spacing means working on tasks spaced across multiple sessions over time instead of massed into a single session.

This approach has been addressed in medical teaching by other studies [8]. For example Kemper et al. performed a controlled trial study among health care professionals delivering content either step by step or within a short time period. The results showed no significant differences concerning completion rate and outcomes, but they did not analyze details of the usage pattern [9].

### Aims

To support the spacing effect we investigated whether continuous learner activation giving access to VPs step by step would change the usage pattern and lower the peak of first time usage before the exam.

For our setting we analyzed the differences of two groups (spaced vs. continuous access) concerning

• Usage pattern including number of sessions and session duration

• Score on the questions integrated into the VPs

• Results in the intermediate exam.

## Methods

### Participants

For the summer term a total number of n= 207 were enrolled in the pediatric course. They were randomly divided in Group A (n=107) and Group B (n=100) by the dean’s office. These two groups were comparable concerning age, gender and prior knowledge level (i.e. courses they have completed so far).

### Instruments: implementation of the pediatric E-learning module

#### The E-learning module consisted of the following components

1. “Age guessing” (4 video-based exercises training the skill of estimating the age of children between the age of 3 months and 5 years according to their psychomotoric development)

2. Clinical skills online (Cliso) module consisting of 3 VPs (video-based) and background information about pediatric clinical examination skills as preparation for the internship [10]

3. 7 obligatory, exam relevant VPs (common and relevant diseases)

4. 6 optional VPs (incentive: certificate when completing all 13 VPs, intended for students with a special interest in pediatrics)

5. “Look & Choose” (52 characteristic picture-based exercises dealing with common and relevant signs and symptoms in childhood, e.g. skin rashes, dysmorphic signs)

6. "Emergency VP" - Emergency medicine VP to be completed within 24 hours during the mini-internship

Age-Guessing and Cliso were intended to be completed as preparation before entering the pediatric course. Look & Choose was intended to be completed before starting the mini-internship (bedside teaching).

Each learning unit was created by a team of at least one physician supported by a student. The content was reviewed by experts in the clinical field. Before the module started, it was tested and reviewed/evaluated by 15 students. It was designed to match and complement the learning objectives of the pediatric course of the LMU faculty.

The E-learning unit was created with the case-based learning system Casus®, which is suitable to teach clinical reasoning skills [11]. The player allows students to access the VPs and answer the questions. The system is linear with a sequential arrangement of screencards composed of text, multimedia elements, expert comments and questions with immediate quantitative (score) and qualitative (detailed answer comment) feedback. The question formats implemented for this course were multiple and single choice, freetext and mapping questions. For Age guessing and Look & Choose exclusively long text answers, which are not rated by the system, have been used.

The learning objectives covered by the VPs were included on the last screencard.

Although the VPs can be accessed as often as desired, the course setting did not allow students to reset the session in order to answer the questions again or change given answers. The time spent on a VP is cumulative throughout the sessions.

We estimated that students would spend an overall time of about 18 hours working on the E-learning module. This was also stated in the university calendar. This estimation was based on our experience with the integration of VPs in other content domains.

#### Setting

The pediatric curriculum at the medical faculty of the LMU is structured as a 4 weeks academic course including a 4-day mini-internship at the end. It is held twice per term during the 4th year of medical school. Two groups (A and B) go through this course each term subsequently.

#### The 4 weeks course comprises

• 25 lectures (45 min each; all students)

• 7 seminars (90 min each; groups of 20 students)

• 8 tutorials/problem based learning (90 min each; groups of 10 – 15 students)

• 4 days mini-internship (students are assigned to a pediatric ward and practice history taking and examination under tutorial supervision)

• Intermediate written exam at the end of week 3 before the beginning of the mini-internship consisting of 40 open questions (maximum: 40 points). The exam is based on all course components (lectures, tutorials, seminars, internship and VPs).

• Final exam at the end of the term consisting of 40 MC questions (maximum: 40 points). For this study the results of the final exams were not included due to the different time spans between the intermediate exams and the final exam for the two groups.

**Figure 1:**
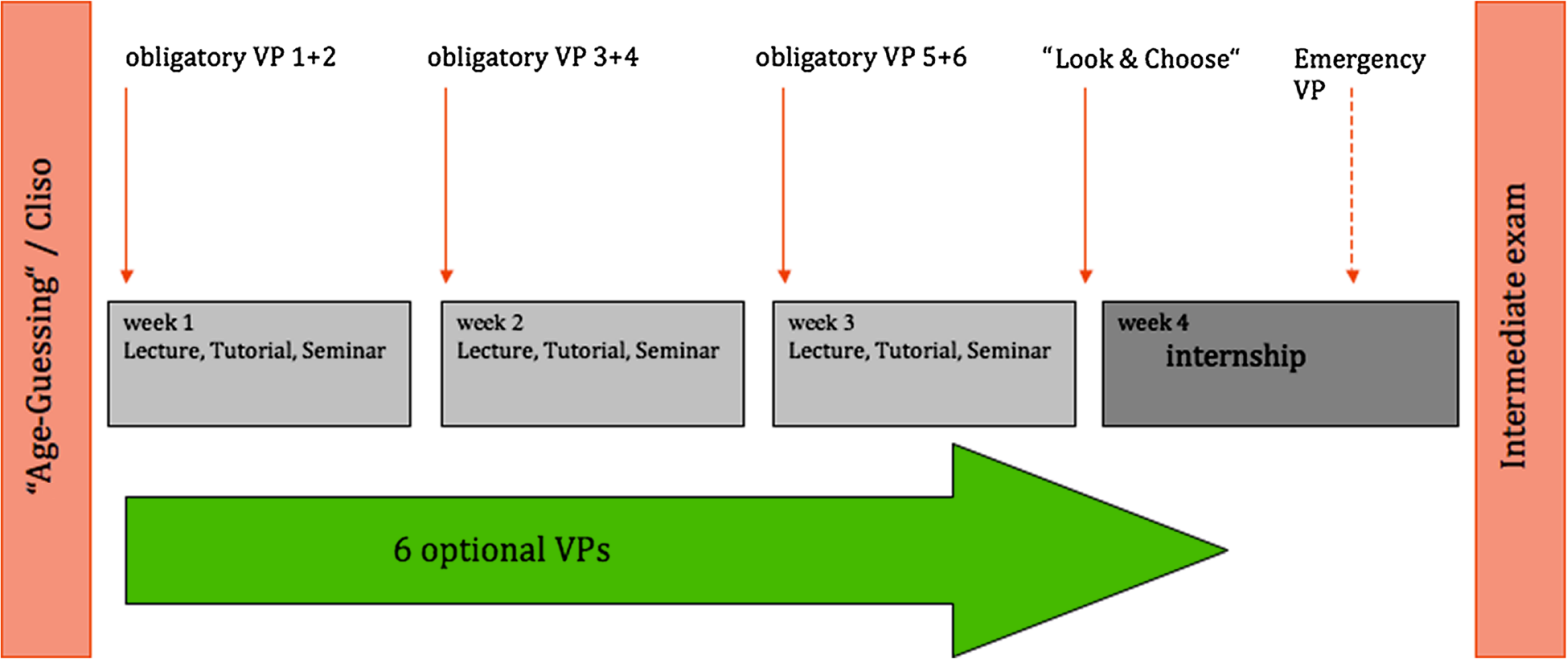
Structure of the pediatric course.

An overview about the course is given in Figure 1.

The pediatric course is part of the so-called “periods of life” term, combined with courses in geriatric medicine, rehabilitation, family medicine and obstetrics/gynecology. When students enter this term they have already completed their basic clinical studies including internal medicine and surgery.

The new E-learning module was introduced into the pediatric curriculum during summer term 2009. Students had to register at the Virtual University of Bavaria (vhb) to obtain an account and could then access the modules.

Group A took the 4 week pediatric course during April/May, Group B during June.

Both groups were asked to prepare for the course by working through the clinical skills module (Cliso) and Age-Guessing. For Group A, the obligatory VPs were presented in a timed order. Every week, two VPs were sequentially activated. This was announced at the beginning of the course, but the course participants did not get any further notification during the course. Students were allowed to work in groups, but we did not explicitly encourage them to do so. There was no extra organizing effort on the tutor side, because the spaced activation could be easily done in the course administration tool of the Casus® system.

For Group B, all obligatory VPs were available from the beginning of the course. But they were also instructed to work on two VPs per week.

The optional VPs as well as the Look & Choose modules were available throughout the course for both groups.

#### Analysis of data

To assess the usage pattern we analyzed the logdata of the sessions in Casus concerning VPs opened, day on which a session was started, duration of session in minutes and score for each answer. A session was counted if at least 50% of the screencards had been completed. This cut-off was arbitrarily set and was based on the curricular approach, where students have to complete at least 50% of cards to get credit for an activity.

We tested for normal distribution using the Shapiro-Wilk test. As all three data sources - VP session duration, scores on answers and results of the intermediate exam - were nearly normally distributed, we applied two-sided t-tests for independent sample-size (level of significance 0.05) to detect differences between group A and B within each data source. The power of the study was assessed 0.99.

The data analysis was done with SPSS, Version 19.

### Ethical aspects

The data used for this study have been made anonymous before analysis. Course participants gave consent for the storing and evaluation of session-related data upon registering for the course. This approach was in accordance with the local rules of the regulatory board and therefore an ethical approval was not required for this study.

## Results

### Usage pattern

In Group A 99 (92.5%) and in Group B 98 (98%) students obtained an account for Casus and started to work on at least one VP. Table 1 summarizes the number of VP sessions for all components of the E-learning module. The descriptive analysis showed no difference in the number of obligatory VP sessions for the two groups (spaced vs. non-spaced).

**Table 1:** Session numbers

**Component**	**A (n=99)**	**B (n=98)**
Age guessing	90 (90.9%)	79 (80.6%)
Cliso	76.3 (77.1%)	80 (81.6%)
Obligatory VPs	98.2 (99.2%)	96.5 (98.5%)
Optional VPs	54.8 (54.4%)	55.2 (58.3%)
Look & Choose	67 (67.7%)	74 (75.5%)
Emergency VP	92 (92.9%)	84 (85.7%)

Table 1 Number of sessions for each component of the module. Included are sessions in which students have opened at least 50% of the screen cards.

**Figure 2:**
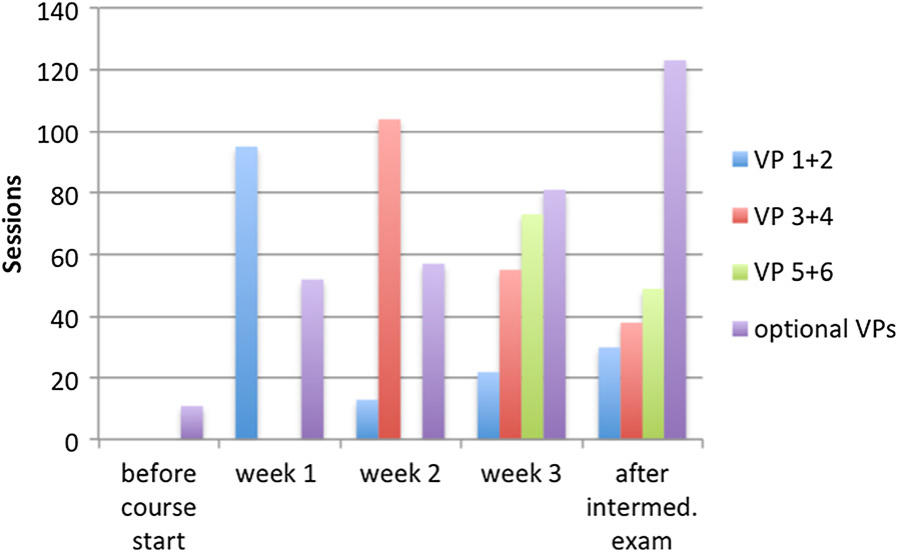
Weekly VP sessions for Group A (spaced) before and after the intermediate exam.

The following figures show the number of new VP sessions before, during and after the three weeks course. Students of Group A show a peak of sessions of the obligatory VPs in the week in which the VPs have initially been activated (VP1 and 2 in week one, VP 3 and 4 in week 2 and VP 5 and 6 in week 3). Figure 2 shows the usage pattern of group A.

**Figure 3:**
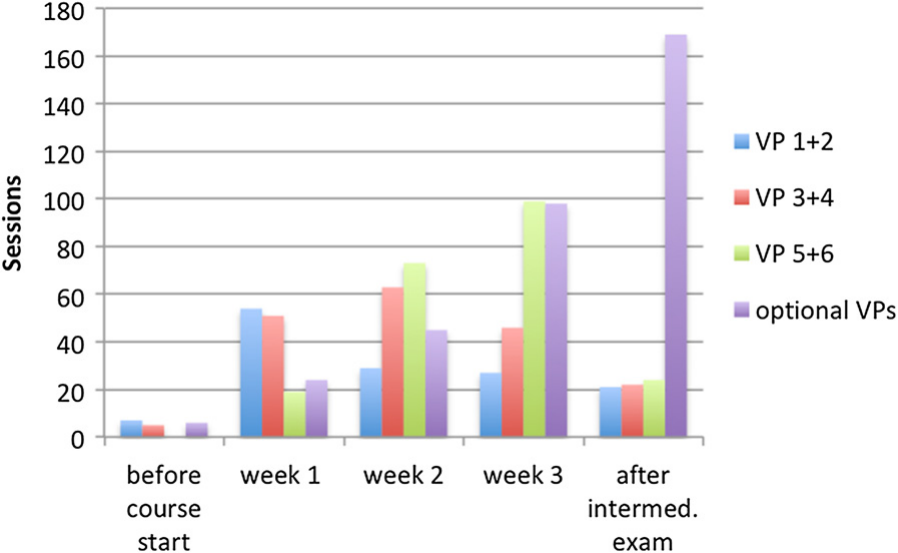
Weekly VP sessions for Group B (no spacing) before and after the intermediate exam.

The usage pattern for Group B is shown in Figure 3.

The number of optional VP sessions increased for both groups continuously from the course beginning until the final exam.

Table 2 gives an overview of the mean values of the obligatory VP session durations. A slightly higher time on task can be seen for Group A, except for VP 6, but none of these differences are significant.

**Table 2:** Time spent on the VPs

**VPs**	**Group A**	**Repeated**	**Group B**	**Repeated**	**T-test: p-value**
	**Time**	**VPs A**	**Time**	**VPs B**	
VP 1	34.6 min	15	31.4 min	6	0.364
	SD: 25.8	(36.8 min)	SD: 21.2	(33.4 min)	
VP 2	44.0 min	14	41.4 min	5	0.522
	SD: 29.2	(47.6 min)	SD: 25.5	(43.4 min)	
VP 3	33.3 min	14	30.6 min	4	0,473
	SD: 28.3	(34.7 min)	SD: 21.7	(31.1 min)	
VP 4	30.2 min	13	30.2 min	3	0.979
	SD: 23.0	(32.5 min)	SD: 19.9	(30.4 min)	
VP 5	25.8 min	16	27.6 min	2	0.533
	SD: 17.4	(29.7 min)	SD: 19.9	(28.2 min)	
VP 6	37.8 min	15	39.4 min	2	0.696
	SD: 18.2	(40.6 min)	SD: 30.5	(40.6 min)	
Repeated VPs		n=87		n=22	

Table 2 Mean values of VP session duration in minutes for the obligatory VPs in Group A and B without the sessions that have been repeated before the final exam. In brackets the overall time spent on the VP including repeated sessions is stated. SD=Standard deviation.

When comparing the numbers of VP sessions that have been repeated after the intermediate exam and before the final exam, there is a considerable difference between Group A (87 repeated sessions) and Group B (22 repeated sessions). This fact leads to a longer session time. The average session time without these re-visited sessions, can be seen in Table 2 as numbers in brackets. Interestingly the average time on task for group A is higher for VPs 1–3, equal for VP 4 and lower for VPs 5 and 6 compared to group B, but none of these differences are significant. For all other optional VPs and modules there were no significant differences in session time between the two groups.

### Overall score on answers integrated into the VPs

For the Cliso VPs, the obligatory and the optional VPs there was no significant difference concerning the percentage of correct answers (p-values: Cliso=0.684, obligatory VPs=0.300, optional VPs=0.218). For Age guessing and Look & Choose the answers of the students have not been rated.

### Results of the intermediate exam

Comparing the results of the intermediate exams there were no significant differences (p-value=0.594) between Group A (mean value 35.5 points, SD=2.8) and B (mean value 35.9 points, SD=3.6).

## Discussion

For the creation of the pediatric E-learning module, we followed basic, but important recommendations such as easy to use software and providing a self-contained peer reviewed learning unit that covers the curricular objectives and the objectives assessed in the exams [2,4,12].

Comparing the high rate of VP sessions especially of the optional VPs with similar integration strategies experienced in earlier studies we conclude that the integration and motivation of students was successful [4].

Also in accordance with the findings of other studies are the high completion rates for the obligatory VPs, with more than 95% in both groups.

### Usage pattern

The usage pattern (see chart 3) shows that when activating VPs each week, students tend to work on the new VPs during this week, which leads to a more balanced usage pattern. Moreover this chart indicates that also the work on the optional VPs, that have been accessible throughout the course for both groups, is more balanced, with a lower peak before the exam.

In accordance with the study results of Kemper et al [10] we also could not see any significant differences between the two groups with the different mode of activation concerning the number of obligatory VPs they have completed. However, we could see a slight but non-significant difference in the duration of these VP sessions. 5 of the 6 VPs were completed by the students of Group A with a longer duration than for Group B. When recalculating these times without the VPs which have been repeated before the final exam, the time on task is higher for VPs 1–3, equal for VP 4 and lower for VPs 5 and 6 compared to Group B. This could mean that the effect of spacing initially also has a positive effect on the session duration, but levels out later in the course.

Interestingly both groups showed no significant difference in their score of the questions integrated into the VPs. This is remarkable because one could assume that if students complete the VPs towards the end of their course, their knowledge about the topic should be higher.

Further studies have to be implemented to investigate this on a more detailed level.

Group A shows a significantly higher repetition rate compared to Group B, which is most likely due to the fact that there is a much longer time between their intermediate exam and the final exam than for Group B. This was an influencing factor for our study we could not eliminate and was a limitation of the study. However, this showed us that the setting for Group A would be optimal within the given conditions. Students in this group had the spaced activation that motivated them to work on the VPs regularly throughout the course. Additionally they had quite a long period of time between the end of their course and their final exam, which made them go back to the VPs before their exam and repeat them, so they presumably have a better long-term knowledge retention.

## Conclusions

Our study showed that a spaced activation led to a more balanced usage pattern with a lower peak of VP sessions right before the intermediate exam. Further studies will have to investigate in detail whether a spaced activation has a significant effect on the session duration and whether this effect is the same throughout the course.

The effect of spacing and repetition has until recently only been addressed by a few studies in the area of internet based teaching in general [8]. However, this is an important aspect when integrating E-learning modules, such as VPs, into a medical curriculum with mixed instructional methods.

In the pediatric course we will continue to implement the spaced activation of VPs for both groups, because of the more balanced usage pattern. Until now students could only repeat VPs in a non-interactive way, meaning they could not answer the questions again. But as shown by Schmidmaier et al it is more efficient to repeat testing instead of learning [6]. So we will allow students of both groups to reset their VP sessions to repeat them in an interactive way.

Additionally, we would like to conduct a future study that will focus on the effect of spacing, as implemented for Group A, on long-term knowledge retention.

## Competing interests

IH und MF hold shares of the Casus-software company Instruct. Otherwise the authors declare that they have no financial nor non-financial competing interests.

## Authors’ contributions

EM drafted the manuscript and contributed significantly to the conception, design and implementation of the study. IH drafted the manuscript and contributed significantly to the conception, design and implementation of the study. AM contributed substantially to the conception, design and implementation of the study. JH contributed substantially to the data collection, analysis and interpretation. MRF contributed substantially to the conception and design of the study and gave major didactical input. All authors have revised the manuscript critically and given final approval.

## Pre-publication history

The pre-publication history for this paper can be accessed here:

http://www.biomedcentral.com/1472-6920/13/45/prepub
